# Scientists and Bioethics Councils

**DOI:** 10.1371/journal.pbio.0020177

**Published:** 2004-06-15

**Authors:** Anne McLaren

## Abstract

In response to the Blackburn and Rowley essay on the President's Council on Bioethics, several thought-provoking opinions on ethical challenges in biomedical research are expressed by prominent stakeholders

I read with interest the article in a recent issue of *PLoS Biology* by Elizabeth Blackburn and Janet Rowley, two of the scientific members of President Bush's Council on Bioethics. Invited by the President to serve on this Council, they say that it was ‘a difficult invitation to accept’. Maybe, but that they did accept the invitation is to be applauded. As the Council's report ‘Monitoring Stem Cell Research’ states, ‘fairness in ethical evaluation and judgment depends on … fair and accurate description of the relevant facts of the case at hand’. In other (fewer) words, sound ethics requires a solid base in sound science. It is crucial that any bioethics committee or council made up of ten to twenty members should include at least two or three scientists broadly acquainted with the field in general, and with recent published findings. I was only sorry to read that Elizabeth Blackburn (who works in California but is a Fellow of the Royal Society, the United Kingdom Academy of Sciences) had her Council term terminated by White House directive on February 27, 2004.

Of course, any bioethics committee or council (and I have served on several such, both in the UK and elsewhere in Europe) is likely also to include philosophers, lawyers, theologians, sociologists, and probably ‘lay’ people of appropriate interests. The scientists may well find that other members of the group have ‘strong opposing views’ on ethical issues, as well as on the costs and benefits of technologies arising from biomedical research. Elizabeth Blackburn and Janet Rowley were assured, both by Leon Kass, the chairman of the Council, and by President Bush himself, that their voices would be heard and integrated into the Council statements. It is therefore disappointing to learn that, in the ‘Beyond Therapy’ report (which I have not yet read), their requests for revision of certain aspects was declined. Were they not offered the option of a brief minority report? It would be expected in such circumstances that dissenting opinions would be recorded (as was done, for example, in the 1984 UK report by the Committee on Human Fertilisation and Embryology chaired by Mary Warnock (1984), and in some of the Opinions offered by the European Group of Ethics to the European Commission). This would be particularly appropriate, and indeed essential, when recommendations are put forward.

The ‘Monitoring Stem Cells Research’ report (which I have read) contains no recommendations, but includes a rather comprehensive survey of the various ethical positions relating to human embryonic stem cell research, a historical account of the development up to the present time of federal law and policy, and a chapter on recent (almost entirely United States) developments in human stem cell research and therapy. The scientists must have contributed substantially to this section of the report. Emphasis is put on the need for research on both adult and embryonic stem cells, since at present there is no way to assess which approach has the more promising therapeutic potential for which diseases. Some funding figures are given: on human embryonic stem cell research the US National Institutes of Health spent $10.7 million in 2002 and $17 million in 2003, with an estimated total spent by US companies of $70 million, while in the same two years the National Institutes of Health spent $170 million in 2002 and $181.5 million in 2003 on adult stem cell research. However, it is not obvious that there are any US scientists wanting to work on human embryonic stem cells within the constraints of US federal funding who are prevented from doing so by lack of money.

To my mind, the major deficiency in the ‘Monitoring Stem Cells Research’ report is the almost complete lack of reference to what Elizabeth Blackburn and Janet Rowley correctly call ‘years of rigorous and careful research in animal models’. Some mention is made of experiments with human embryonic stem cells in immunologically handicapped mice, but in any such model both the stem cells and the mice are difficult to work with. Much of the science-based optimism that human embryonic stem cells may eventually prove of therapeutic value springs from the results of experiments with mouse embryonic stem cells in intact mice. Curiously, only a single such experiment is cited: an impressive but somewhat recondite piece of work from Jaenisch's laboratory (Rideout et al. 2002), using cloned and genetically modified mouse embryonic stem cells to treat a form of mouse hepatitis. A wider consideration of work on animal models, together with some emphasis on the potential use of human embryonic stem cells for toxicity testing and drug design by pharmaceutical companies, is in part what Elizabeth Blackburn and Janet Rowley believe ‘would help the public and scientists better assess the content of the report’. If they requested inclusion of such material, it is unfortunate that their requests were declined.
